# F1 Domain of the *Leishmania (Leishmania) donovani* Nucleoside Hydrolase Promotes a Th1 Response in *Leishmania (Leishmania) infantum* Cured Patients and in Asymptomatic Individuals Living in an Endemic Area of Leishmaniasis

**DOI:** 10.3389/fimmu.2017.00750

**Published:** 2017-07-12

**Authors:** Eugenia Carrillo, Laura Fernandez, Ana Victoria Ibarra-Meneses, Micheli L. B. Santos, Dirlei Nico, Paula M. de Luca, Cristiane Bani Correa, Roque Pacheco de Almeida, Javier Moreno, Clarisa B. Palatnik-de-Sousa

**Affiliations:** ^1^WHO Collaborating Centre for Leishmaniasis, Centro Nacional de Microbiologia, Instituto de Salud Carlos III, Madrid, Spain; ^2^Departamento de Medicina, Hospital Universitário, Universidade Federal de Sergipe, Aracaju, Brazil; ^3^Instituto de Microbiologia Paulo de Góes, Universidade Federal do Rio de Janeiro, Rio de Janeiro, Brazil; ^4^Laboratório de Imunoparasitologia, Instituto Oswaldo Cruz, Rio de Janeiro, Brazil; ^5^Departamento de Morfologia, Universidade Federal de Sergipe, Aracaju, Brazil; ^6^Instituto Nacional de Ciência e Tecnologia de Investigação em Imunologia, São Paulo, Brazil

**Keywords:** human visceral leishmaniasis, cutaneous leishmaniasis, nucleoside hydrolase, B cell epitopes, T cell epitopes, adaptive immunity

## Abstract

The *Leishmania (Leishmania) donovani* nucleoside hydrolase NH36 is the main antigen of the Leishmune^®^ vaccine and one of the promising candidates for vaccination against visceral leishmaniasis. The antigenicity of the N-terminal (F1), the central (F2), or the C-terminal recombinant domain (F3) of NH36 was evaluated using peripheral blood mononuclear cells (PBMC) from individuals infected with *L. (L.) infantum* from an endemic area of visceral leishmaniasis of Spain. Both NH36 and F1 domains significantly increased the PBMC proliferation stimulation index of cured patients and infected asymptomatic individuals compared to healthy controls. Moreover, F1 induced a 19% higher proliferative response than NH36 in asymptomatic exposed subjects. In addition, in patients cured from visceral leishmaniasis, proliferation in response to NH36 and F1 was accompanied by a significant increase of IFN-γ and TNF-α secretion, which was 42–43% higher, in response to F1 than to NH36. The interleukin 17 (IL-17) secretion was stronger in asymptomatic subjects, in response to F1, as well as in cured cutaneous leishmaniasis after NH36 stimulation. While no IL-10 secretion was determined by F1, a granzyme B increase was detected in supernatants from cured patients after stimulation with either NH36 or F1. These data demonstrate that F1 is the domain of NH36 that induces a recall cellular response in individuals with acquired resistance to the infection by *L. (L.) infantum*. In addition, F1 and NH36 discriminated the IgG3 humoral response in patients with active visceral leishmaniasis due to *L. (L.) donovani* (Ethiopia) and *L. (L.) infantum* (Spain) from that of endemic and non-endemic area controls. NH36 showed higher reactivity with sera from *L. (L.) donovani*-infected individuals, indicating species specificity. We conclude that the F1 domain, previously characterized as an inducer of the Th1 and Th17 responses in cured/exposed patients infected with *L. (L.) infantum chagasi*, may also be involved in the generation of a protective response against *L. (L.) infantum* and represents a potential vaccine candidate for the control of human leishmaniasis alone, or in combination with other HLA epitopes/antigens.

## Introduction

Leishmaniasis is a vector-born infectious disease caused by a group of protozoan parasites of the genus *Leishmania*. *Leishmania (L.) infantum* infection is endemic in parts of the Mediterranean basin, Asia, and Central and South America, where it is widespread and represents a serious public health problem. In Spain, the infection can be either asymptomatic or manifest in both cutaneous (CL) and visceral (VL) forms (1, 2), the last one being fatal if untreated (3).

While vaccination has been considered as a very effective tool for the eradication of canine visceral leishmaniasis (canine VL) (4, 5) and four veterinary vaccines were already licensed (6–9), there is still no vaccine available for use in humans. In Europe, there are two veterinary vaccines, one composed of the 54 kDa native excreted/secreted protein (Canileish) (6) and the other a chimera recombinant multiprotein protein Q, which contains the acidic ribosomal proteins Lip2a, Lip2b, P0, and histone H2A (7) (Latifend). In Brazil, the FML glycoprotein extract of *L. (L.) donovani* (Leishmune^®^) (8–11) was the first commercial vaccine in the world licensed in 2003. After that, the A2 recombinant protein of *L. (L.) infantum chagasi* (Leish-Tec^®^) (12) was also licensed in Brazil, in 2007 (13).

While untreated VL patients showed a failure in the T-cell proliferative response to leishmanial antigens, recovery from disease and resolution of infection correlate with the induction of a memory Th1 response in cured/exposed asymptomatic *Leishmania*-infected individuals (14, 15). The ability of vaccine candidates to elicit a Th1 response in infected hosts is indicative of the respective antigen presentation during infection and its potential to generate protection, if used as a component in a vaccine formulation. Many *Leishmania* antigens have been assayed as vaccine candidates in animal models with variable success (16–18). The main antigen of Leishmune^®^ is the *L. (L.) donovani* nucleoside hydrolase (NH36) (8). Vaccination with NH36 DNA has proven to induce protection against murine cutaneous and visceral leishmaniasis (19–25) as well as against canine VL (26, 27) mediated by a CD4^+^ T-cell-dependent interferon (IFN)-γ response (21). Furthermore, the N-terminal domain (F1, amino acids 1–103), the central domain (F2, amino acids 104–198), or the C-terminal recombinant domain (F3, amino acids 199–314) of NH36 have been tested as vaccine candidates against *L. (L.) infantum chagasi* challenge, and F1- and F3-vaccinated mice induced increased levels of IFN-γ and tumor necrosis factor (TNF) (22). Protection in mice against *L. (L.) infantum chagasi* infection was mediated by a CD4^+^ T-cell response to the F3 domain. The intradermal response to leishmanial antigen (IDR) and an increased tumor necrosis factor alpha (TNF-α) secretion were the strong correlates of this F3 domain-induced protection (22).

In contrast, prevention and cure of cutaneous leishmaniasis caused by *L. (L.) amazonensis* involved a CD8^+^ T-cell response to the F1 domain, besides the CD4^+^ T-cell response to the F3 domain (23, 24). We recently described that the *in silico-*predicted epitopes of *L. (L.) donovani*-NH36 are highly conserved in the nucleoside hydrolase of *L. amazonensis* (24). This justifies the high cross-protection obtained against *L. (L.) amazonensis* infection of mice, after vaccination with F1 and F3 (23–25). Furthermore, a recombinant chimera composed of the F1 and F3 domains cloned in tandem optimized that vaccine efficacy (25).

The *in silico* analysis revealed that the predicted epitopes of NH36 for HLA-DR and HLA-A and B histocompatibility molecules of humans (28) slightly differ from those described for MHC molecules of mice (22, 24). In spite of that difference, the F1 domain, which induced a CD8^+^ T-cell-mediated protection against cutaneous leishmaniasis of mice (22, 24, 25), shares important epitopes for the generation of a Th1 and CD8^+^ T-cell responses of human VL patients infected with *L. (L.) infantum chagasi* from Sergipe, Brazil (28). However, while *L. (L.) infantum chagasi* is the etiological agent of VL in America, the disease is caused by *L. (L.) infantum* in Europe. We recently showed that the epitopes of NH36 recognized by the histocompatibility molecules of humans are highly conserved among species of the *Leishmania* genus that cause both the visceral and the cutaneous forms of leishmaniasis in all continents (28). In addition, we identified the NH36 domains, which are the markers of VL resistance, in asymptomatic and cured subjects and of clinical VL outcomes, in a Brazilian endemic area of *L. (L.) infantum chagasi* infection (28).

As a further step in the development of a universal vaccine, able to protect against human VL in Europe and America, the identification of the main immunogenic domains of *L. (L.) donovani*-NH36 recognized by human asymptomatic and cured patients, infected by *L. (L.) infantum*, in Europe is necessary. In addition, it is important to identify if the domains of NH36 are recognized differently by patients infected by *L. (L.) infantum*, which develop the visceral, or the cutaneous form of leishmaniasis (1, 2).

In this investigation, we report the recognition of NH36 and its domains by peripheral blood mononuclear cells (PBMC) of *Leishmania*-exposed and cured individuals from an *L. (L.) infantum* endemic area of Spain. The proliferation to NH36 and F1 domains was accompanied by a statistical increase in IFN-γ, TNF-α, interleukin (IL)-17, and granzyme secretion. Our data demonstrate that F1 is a potential candidate for the development of a synthetic human vaccine formulation against VL in Europe and America.

## Materials and Methods

### Ethical Statement

This study was approved by the Hospital de Fuenlabrada (Madrid) Ethics and Research Committee (APR 12–65 and APR 14–64). In addition, the protocols for the collection of sera samples of healthy blood donors of the Hospital Universitário Clementino Fraga Filho (HUCFFo)-Blood Transfusion service of the Universidade Federal do Rio de Janeiro (Rio de Janeiro, Brazil) were approved by the Comité de Ética em Pesquisa of the HUCFFo-Faculdade de Medicina according to the GM-MS 158-2016 regulation of the Brazilian Ministry of Health. All participants gave their written informed consent.

### Blood and Sera Samples

Subjects included in this study were residents of Fuenlabrada, an *L. (L.) infantum* postoutbreak area of Madrid, Spain. Blood samples were obtained from the patients of the Hospital de Fuenlabrada, from 2013 to 2015, and forwarded to the Instituto de Salud Carlos III, where the immunological assays were performed. Patients clinically diagnosed with either visceral (VL) or cutaneous leishmaniasis (CL) were treated with liposomal amphotericin B or with intralesional meglumine antimoniate, respectively. Three months after the end of the treatment, cure was confirmed in 16 patients. Their PBMC were used for a preliminary screening of antigenicity in a cell lymphoproliferation assay. Further lymphoproliferation analysis and quantification of cytokines secreted to the supernatants were performed in antigen-stimulated PBMC from additional 10 patients cured from visceral leishmaniasis (CVL) and eight individuals cured from cutaneous leishmaniasis (CCL).

In addition, healthy blood donors of the Hospital de Fuenlabrada-Blood Bank (*n* = 41) were screened according to their response to the soluble *Leishmania* antigen (SLA) in an *in vitro* PBMC proliferation assay. Among them, 11 donors reacted positively and were considered as *Leishmania*-infected asymptomatic subjects. Moreover, 30 subjects with negative reaction in the SLA proliferation assay were considered as endemic area normal healthy controls and were randomly distributed for lymphoproliferative assays (*n* = 17) and cytokine analyses (*n* = 13).

For ELISA, we analyzed sera samples of 33 *L. (L.) infantum*-VL cases from Spain. Their clinical status was confirmed by positive results in the direct agglutination test results (DAT) and rK39 serological assays. Furthermore, 24 cases of *L. (L.) donovani-*VL infection from Amhara, Ethiopia, were confirmed by their positive DAT results and negative delayed type of hypersensitivity (DTH) reactions to leishmanial antigen (29). Asymptomatic subjects, in contrast, showed DAT-negative and DTH-positive responses. All the sera samples of *L. (L.) infantum*- and *L. (L.) donovani*-infected individuals belong to the Instituto de Salud Carlos III-WHO Collaborating Center for Leishmaniasis-Biobank. This biobank is registered in the National Registry of Biobanks with reference C.000898 (in accordance with the regulation established by RD1716/2011 Spain).

We also included, as controls, 25 sera samples of healthy blood donors from the Hospital de Fuenlabrada-Blood Bank and 23 sera of healthy blood donors from Rio de Janeiro, a VL non-endemic area of Brazil (NEC), collected at the HUCFFo-Blood Transfusion service, who tested negatively for hepatitis B and C, HIV, shiphylis, HTV, and Chagas disease.

### Antigens

Soluble *Leishmania* antigen (SLA) was obtained from a stationary phase-promastigote culture of *L. (L.) infantum* (JPC strain, MCAN/ES/98/LLM-722). Briefly, promastigotes were washed in PBS and centrifuged at 1,000 *g* for 20 min at 4°C. The *Leishmania* pellet was suspended in lysis buffer (50 mM Tris/5 mM EDTA/HCl, pH 7), submitted to a freeze and thaw procedure, and sonicated and centrifuged at 27,000 *g* for 4 h at 4°C. The supernatant was aliquoted in sterile conditions and stored at −20°C. Protein concentration in the extract was determined using the Pierce BCA Protein Assay Kit (Thermo Scientific, USA) following the manufacturer’s instructions.

Nucleotide and amino acid sequence data of NH36 are available in the EMBL, GenBank™, and DDJB databases under the accession codes AY007193 and AAG02281.1, respectively, and in the SWISS-PROT database under the accession code Q8WQX2. The recombinant NH36 antigen, composed of a sequence of 314 amino acids, i.e., N-terminal (F1, amino acids 1–103), central (F2, amino acids 104–198), and C-terminal (F3, amino acids 199–314) domains, was cloned in *E. coli* (22) and expressed and purified as modified from the methods of Rodrigues et al. (30) and Saini et al. (31).

Briefly, the protein expression was induced in bacterial suspensions with 1 mM IPTG for 4 h and disrupted by sonication. Pellets were washed with 0.5% CHAPS in 10 mM Tris HCl, pH 8.0, and solubilized in a 20 mM Tris HCl, pH 8.0, 500 mM NaCl, 10% glycerol, 8 M urea-containing buffer. After centrifugation, the proteins in supernatants were purified in a Ni-NTA column using imidazol, refolded with urea and reduced gluthation, and dialyzed. Absence of LPS was confirmed using the “Limulus Amebocyte Lysate” QCL-1000, Lonza kit. The fractions containing the NH36, F1, F2, and F3 recombinant antigens were recovered, dialyzed, and preserved at −80°C. The yield of each expression batch was 4.62 mg for NH36, 5.00 mg for F1, 3.5 mg for F2, and 3.75 mg for F3.

The NH sequences of *L. (L.) infantum* (PubMed databank code: AMP43260.1 and CA F05930.1) were compared with those of NH36 of *L. (L.) donovani* using the PubMed NIH-Blast protein tool.

### PBMC Culture and Cell Proliferation Assay

Peripheral blood mononuclear cells were isolated from heparinized blood by Ficoll-Hypaque gradient (Lymphocytes Isolation Solution, Rafer, UK). PBMC were washed with PBS and suspended in RPMI 1640 (Lonza, Sweden) supplemented with 10% fetal bovine serum and 100 U/ml penicillin/streptomycin (Lonza, Sweden). Aliquots containing 2 × 10^5^ cells/well were distributed in 96-well plates and cultured with supplemented RPMI 1640 medium alone or with the addition of 10 µg/ml of SLA, NH36, F1, F2, or F3 at 37°C in a 5% CO_2_ incubator. Bromodeoxyuridine (BrDU) was added 16 h before the end of the 5-day incubation period, and the cell proliferation was measured by BrDU incorporation using the Cell Proliferation Biotrak ELISA System Kit (GE Healthcare life Science, UK). Absorbance values were read at 450 nm in a microplate photometer (Multiskan FC, Thermo Scientific, USA), and results were expressed as stimulation indexes (SI), which represent the ratio between the absorbance mean of stimulated cells and the absorbance mean of unstimulated cells. Supernatants were collected and stored at −20°C until analyzed for cytokine secretion.

### Multiplex Analysis of Cytokines

The levels of the secreted IFN-γ, TNF-α, IL-10, IL-17A, and granzyme B were measured in 50 µl of the supernatants from SLA, NH36, and F1 (10 µg/ml) stimulated PBMC by flow cytometry analysis using the BD Cytometric Bead Array Human Flex set (Becton & Dickinson Bioscience, USA), as previously described (32). Data were acquired in an FACSCalibur cytometer and analyzed using the FCAP Array software Version 3.0 (Becton & Dickinson Bioscience, USA).

### ELISA

MaxiSorp 96-well plates were coated with 100 µl/well of 20 µg/ml of NH36 and F1 recombinant proteins diluted in carbonate buffer (15 mM Na_2_CO_3_, 28 mM NaHCO_3_, pH 9.6). After blocking with 1% BSA supplemented with PBS for 1 h at 37°C, plates were incubated with 100 µl of 1:200 diluted sera samples in PBS −0.05% Tween 20, for 1 h at 37°C. Plates were subsequently incubated with 1/2,000 diluted mouse antihuman IgG3 HRP conjugate (Invitrogen, USA) for 30 min at 37°C, and immune complexes were revealed with SIGMAFAST OPD peroxidase substrate (Sigma-Aldrich, USA). The absorbance was measured at 492 nm using a microplate photometer (Multiskan FC, Thermo Scientific, USA). The cutoff values were calculated using the receiver operating characteristic (ROC) analysis (33, 34) using the Graphpad Prism6 program and the three categories of accuracy based on AUC–ROC analysis, as described by Solano Gallego et al. (33). We additionally calculated the cutoff values using the Youden index (35–37).

### Statistical Analysis

Data were analyzed using the Mann–Whitney *U* test of the GraphPad Prism version 5.0 software (GraphPad Software, La Jolla, CA, USA). Significance was set at *p* ≤ 0.05.

## Results

### F1 Is the Main Domain of NH36 Recognized by the Lymphoproliferative Response of VL Cured and Asymptomatic Individuals

In VL, the cellular immune response to leishmanial antigens is lost during the active disease but is maintained in the infected asymptomatic subjects and cured individuals. We, therefore, decided to study the lymphoproliferative response of these three populations to the NH36 and its domains, using samples of *L. (L.) infantum*-infected subjects obtained from the endemic area of Spain.

**Figure 1:**
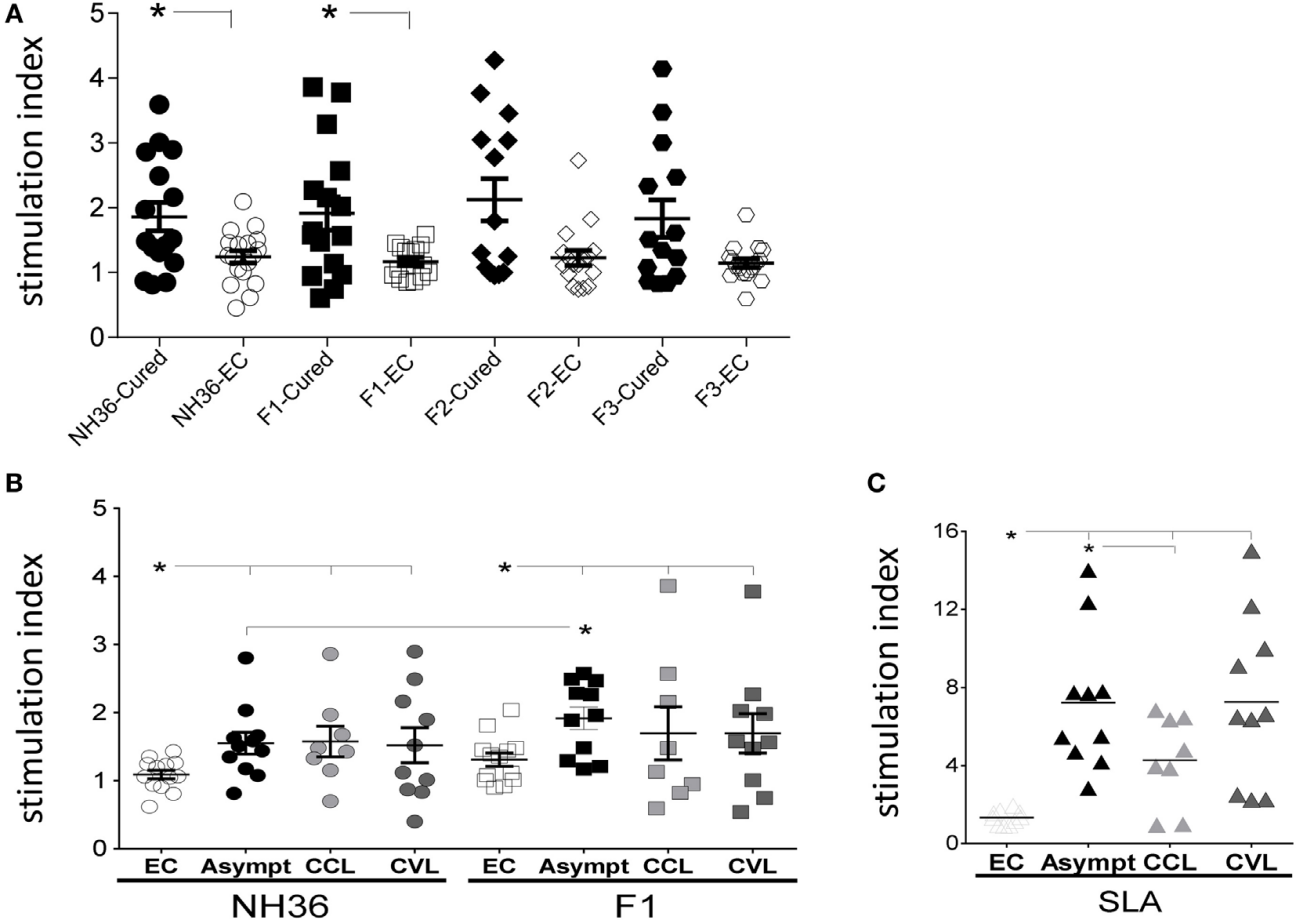
F1 significantly enhances the lymphoproliferative response of cured and asymptomatic visceral leishmaniasis (VL) patients. **(A)** A preliminary screening of the specific proliferation to F1, F2, F3, and NH36 antigens was performed using peripheral blood mononuclear cells from cured VL patients and negative endemic controls (EC) (*n* = 14–16). **(B)** Anti-NH36 and anti-F1 proliferative responses were assessed in EC (*n* = 13), asymptomatic subjects (Asympt) (*n* = 11), cured cutaneous leishmaniasis (CCL) (*n* = 8), and cured VL patients (CVL) (*n* = 10). **(C)** Soluble *Leishmania* antigen (SLA) proliferative responses was assessed in EC (*n* = 10), asymptomatic subjects (Asympt) (*n* = 10), CCL (*n* = 8), and cured VL patients (CVL) (*n* = 10). Each symbol represents one individual; bars represent the mean of the experimental groups. Mann–Whitney test was used to compare clinical groups (*p ≤ 0.05).

An initial screening with NH36, F1, F2, and F3 disclosed that only the NH36 (*p* = 0.042) and F1 (*p* = 0.019) domains induced significant differences between cured leishmaniasis patients and negative controls (Figure 1A). Both NH36 and F1 promoted 1.4-fold increases of their respective SI, which were 30–31% higher in cured patients than in negative controls (Figure 1A).

Based on the cellular immune response results, we decided to focus this study in testing the antigenicity of NH36 and F1 in asymptomatic individuals and patients cured of VL and CL, from the same VL endemic area of Spain. Incubation with either NH36 or F1 induced higher lymphoproliferative responses in PBMC of asymptomatic subjects and cured VL and CL patients than in endemic area controls (Figure 1B). The responses of asymptomatic individuals to NH36 or F1 antigens were not different from those of the cured VL or CL patients. However, it is worth to note that, in the asymptomatic subjects, the F1 domain induced a 19% higher stimulation index than NH36, equivalent to 1.2-fold increase. This result confirmed the antigenic predominance of F1 in acquired resistance to the disease (Figure 1B). We, therefore, concluded that the lymphoproliferative response of cured and asymptomatic subjects against the NH36 antigen is mainly directed toward its F1 immunodominant domain.

Similar to what detected for NH36 and F1 (Figure 1B), we also observed SLA-mediated stimulation in asymptomatic and cured patients (Figure 1C), but with higher SI, as expected for being composed of multiple antigens. In contrast to NH36 and F1, which stimulated PBMC of cured CL, VL, and asymptomatic patients to a similar extent, the SLA complex was preferably recognized by the asymptomatic subjects and cured VL patients (Figure 1C).

### F1 Enhances the Th1 and Th17 Cytokine and Cytotoxic Responses

**Figure 2:**
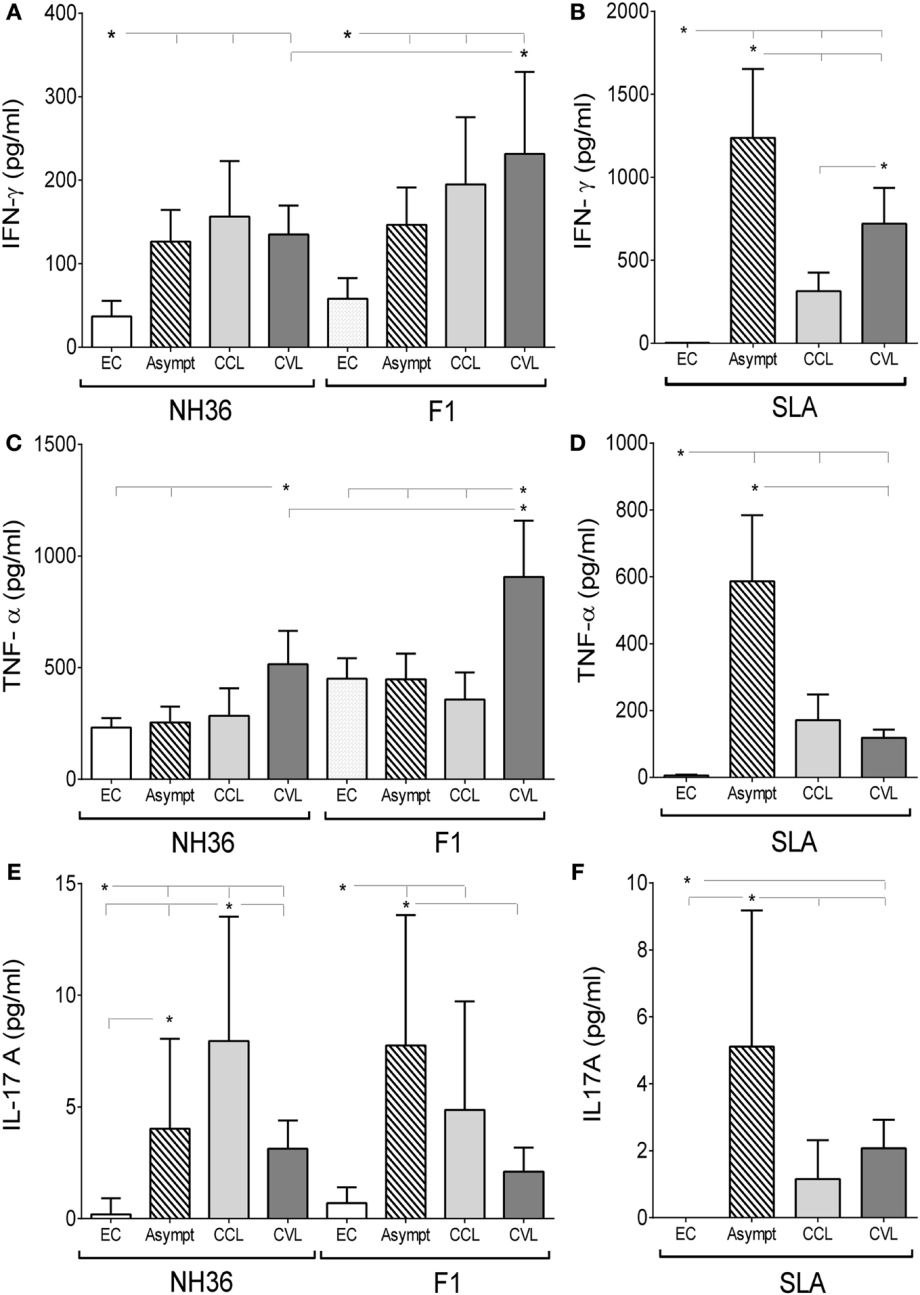
F1 and NH36 enhance the peripheral blood mononuclear cells (PBMC) secretion of interferon (IFN)-γ, tumor necrosis factor alpha (TNF-α), and interleukin (IL)-17A. The secretion of IFN-γ **(A)**, TNF-α **(C)**, and IL-17A **(E)** was assessed in supernatants of NH36- and F1-stimulated PBMC of endemic negative controls (EC, *n* = 13), asymptomatic subjects (Asympt, *n* = 11), cured cutaneous leishmaniasis (CCL, *n* = 8) and cured visceral leishmaniasis (CVL, *n* = 10) patients from visceral leishmaniasis endemic area. Supernatants of PBMC stimulated by the soluble *Leishmania* antigen (SLA) control were also assayed for IFN-γ **(B)**, TNF-α **(D)**, and IL-17A **(F)** secretions. Each symbol represents one individual; bars represent the mean of the experimental groups. Mann–Whitney test was used to compare clinical groups (*p ≤ 0.05).

Regarding the Th1-cytokine response, both NH36 and F1 promoted higher IFN-γ secretion in asymptomatic subjects and cured CL and VL patients than in their respective endemic area controls (*p* < 0.0001) (Figure 2A). The highest IFN-γ secretion was observed in cured VL patients against the F1 domain. In fact, the F1 peptide induced 42% more IFN-γ secretion than NH36 (1.7-fold increase) (Figure 2A). Stimulation with SLA, in contrast, also promoted higher IFN-γ secretion in all groups of patients than in controls (*p* < 0.0001). The highest response to SLA was observed in asymptomatic individuals and, to a lower extent, in cured VL patients (Figure 2B).

As observed for IFN-γ (Figure 2A), a similar performance was observed for TNF-α secretion, which was also more pronounced in cured VL patients, in this case against both the NH36 antigen and the F1 domains (Figure 2C), and was 43% higher in response to F1 than to NH36 (1.8-fold increase). SLA stimulation, in contrast, promoted less TNF-α secretion than the F1 domain (Figure 2C) and a main response observed in asymptomatic subjects (*p* ≤ 0.0001) (Figure 2D).

In addition, IL-17A was secreted by all groups of patients, in response to NH36 (Figure 2E), and by asymptomatic and cured CL patients, in response to F1 (Figure 2F). The highest NH36-specific IL-17A was observed in cured CL patients, while asymptomatic developed the most pronounced response to F1 (Figure 2E). As observed for IFN-γ (Figure 2B) and TNF-α secretion (Figure 2D), SLA stimulation induced a specific increase in IL-17A in asymptomatic individuals. The potency of the NH36 and F1 antigens is remarkable when compared with that of SLA control, which in spite of being composed of many *Leishmania* proteins and PAMPS and being expected to potentiate the cytokine response, promoted a much lower secretion of TNF-α and IL-17A (Figures 2D,F).

**Figure 3:**
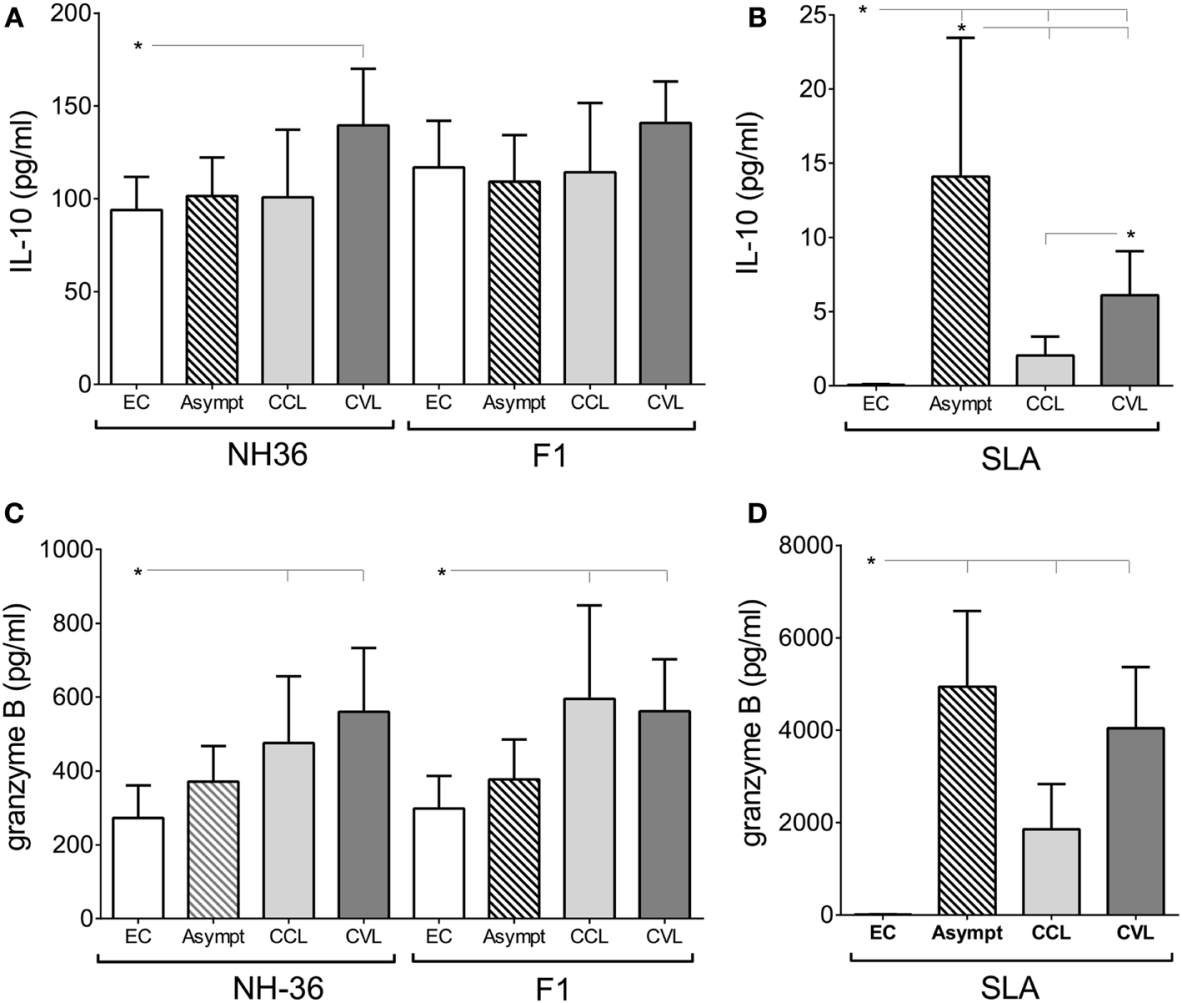
Granzyme B secretion is increased in response to NH36 and F1, while only NH36 induces the increased secretion of interleukin (IL)-10. IL-10 **(A)** and granzyme B **(C)** secretions in response to NH36, and F1, was assessed in stimulated supernatants from endemic negative controls (EC, *n* = 13), asymptomatic subjects (Asympt, *n* = 11), cured cutaneous leishmaniasis (CCL, *n* = 8) and cured visceral leishmaniasis (CVL, *n* = 10) patients from a visceral leishmaniasis area. The control IL-10 **(B)** and granzyme B **(D)** responses induced by soluble *Leishmania* antigen (SLA) were also assayed. Mann–Whitney test was used to compare clinical groups (*p ≤ 0.05).

Regarding the Th2-cytokine response to the antigens, the global antigen-specific IL-10 response was very low. In fact, while no increase in IL-10 secretion was observed against F1, only PBMC of cured VL patients, incubated with NH36, secreted IL-10 (Figure 3A). SLA, in contrast, stimulated IL-10 secretion in all groups of patients (*p* ≤ 0.05) and was predominant in asymptomatic subjects (Figure 3B), as detected for IFN-γ, TNF-α, and IL-17A secretions (Figures 2B,D,F).

Suggesting that NH36 and F1 are capable of triggering a potential Th1 response, the IFN-γ/IL-10 and TNF-α/IL-10 ratios induced by both antigens were higher in asymptomatic individuals and cured CL and VL patients than in the endemic area controls (Table 1). In addition, in response to both antigens, the TNF-α/IL-10 ratios were higher than the IFN-γ/IL-10 ratios (Table 1).

**Table 1:** Superiority of the F1 over the NH36 antigen in interferon (IFN)-γ and tumor necrosis factor alpha (TNF-α)/interleukin (IL)-10 ratios.

Antigens	F1				NH36			
Patients	Endemic controls (EC)	Asym.^a^	Cured cutaneous leishmaniasis (CCL)	CVL	EC	Asym.	CCL	CVL
IFN-γ (pg/ml)	58	147	195	231	37	126	156	135
TNF-α (pg/ml)	451	448	357	906	232	254	285	516
IL-10 (pg/ml)	117	109	114	141	94	102	101	140
IFN-γ/IL-10 ratio	0.5	1.3	1.7	1.6	0.4	1.2	1.6	1
TNF-α/IL-10 ratio	3.9	4.1	3.1	6.4	2.4	2.5	2.8	3.7
IFN-γ/IL-10 ratio % enrichment	20	8	6	38	–	–	–	–
TNF-α/IL-10 ratio % enrichment	38	39	10	42	–	–	–	–

*Calculation was performed according to the following equation = (F1-NH36/F1) values × 100 = protective increment. doi: 10.1371/journal.pntd.0000866.t003*.

*^a^Asymptomatic*.

Moreover, F1 was more potent than NH36 in the development of the IFN-γ/IL-10 and TNF-α/IL-10 ratios, in all groups of patients (Table 1). This means that the capability of inducing an enhancement of IFN-γ/IL-10 and TNF-α/IL-10 ratios is enriched in F1 (Table 1). Calculation of this enrichment increment disclosed that F1 is 38% more potent than NH36 in the induction of IFN-γ/IL-10 by cured VL patients. In addition, F1 is 39 and 42% stronger than NH36 in an increase in the TNF-α/IL-10 ratios in asymptomatic subjects and cured VL patients, respectively (Table 1).

Furthermore, as an indicator of the activity of CD8^+^ T and cytotoxic cells, we measured the secretion of granzyme B. PBMC of cured VL and CL patients stimulated with either NH36 or F1 secreted more granzyme B than negative controls (Figure 3C), indicating the induction of cytotoxic activity by both antigens. SLA stimulation, in contrast, increased the granzyme B secretion from PBMC of the asymptomatic subjects (*p* ≤ 0.0001) and the cured VL (*p* = 0.0075) and CL patients (*p* ≤ 0.0001) (Figure 3D).

Our results disclosed that the F1 domain was more potent than the cognate NH36 protein in the induction of IFN-γ and TNF-α secretions in cured VL patients, did not enhance the secretion of IL-10, and promoted, together with NH36, an increased cytotoxic activity in patients cured from CL and VL. In addition, F1 together with NH36 stimulated the IL-17A secretion by PBMC of asymptomatic individuals.

### NH36 and F1 Are Recognized by IgG3 Antibodies of Patients Infected by *L. (L.) donovani* and *L. (L.) infantum* with Active VL

**Figure 4:**
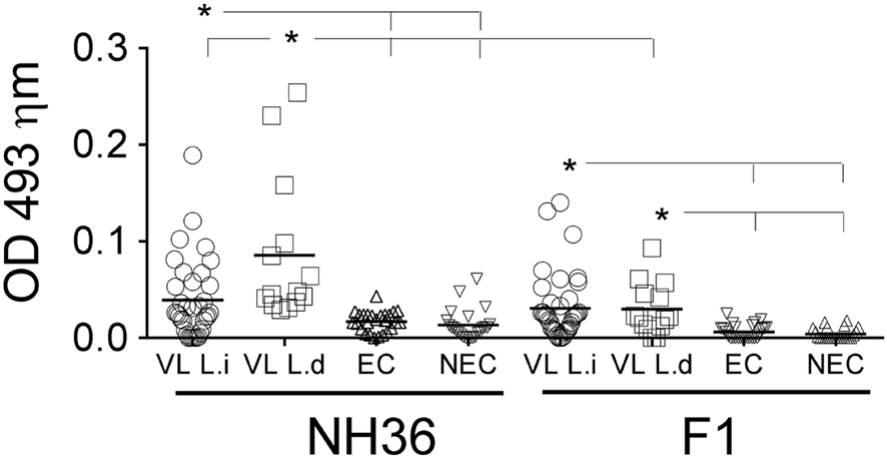
Increased anti-NH36 and anti-F1 IgG3 antibody levels are present in visceral leishmaniasis (VL) infection caused by *L. (L.) donovani* and *L. (L.) infantum*. IgG3 levels in the plasma was assessed by ELISA in European patients with active VL caused by *L. (L.) infantum* (*n* = 33), African patients with active VL caused by *L. (L.) donovani* (*n* = 24), negative controls of endemic area (EC, *n* = 25), and negative controls of a non-endemic area (NEC, *n* = 25). Each symbol represents one individual. Horizontal lines represent the mean of each experimental group. Mann–Whitney test was used to compare clinical groups (*p ≤ 0.05).

We followed our analysis aiming to elucidate the cross-species capabilities of the NH36 antigen and extended the analysis of the humoral response to the antigens, using sera samples of patients from Ethiopia infected with *L. (L.) donovani* and patients from Spain infected with *L. (L.) infantum* (Figure 4).

The IgG3 antibody subtype has been described as a serological marker of VL, and its decrease after successful treatment suggests that it may be a useful tool for diagnosis. The anti-NH36 IgG3 antibodies were 55% higher in patients with active VL from Ethiopia, caused by *L. (L.) donovani*, than in patients infected in Spain with *L. (L.) infantum* (*p* = 0.004) and also higher than in uninfected controls of both non-endemic and endemic areas (*p* ≤ 0.0001 for both) (Figure 4). The IgG3 reactivity to NH36 was also higher in patients infected with *L. (L.) infantum* than in non-endemic and endemic area controls. In contrast, there was no significant difference between the anti-F1 IgG3 reactivity of LV patients infected either by *L. (L.) donovani* (*p* ≤ 0.01) or by *L. (L.) infantum* (*p* ≤ 0.0001), although both were respectively higher than those observed in the non-endemic and endemic control subjects (Figure 4).

Noteworthy, the response of patients infected with *L. (L.) donovani* from Ethiopia was 35% higher against NH36 than against F1 (*p* ≤ 0.01) (Figure 4).

**Figure 5:**
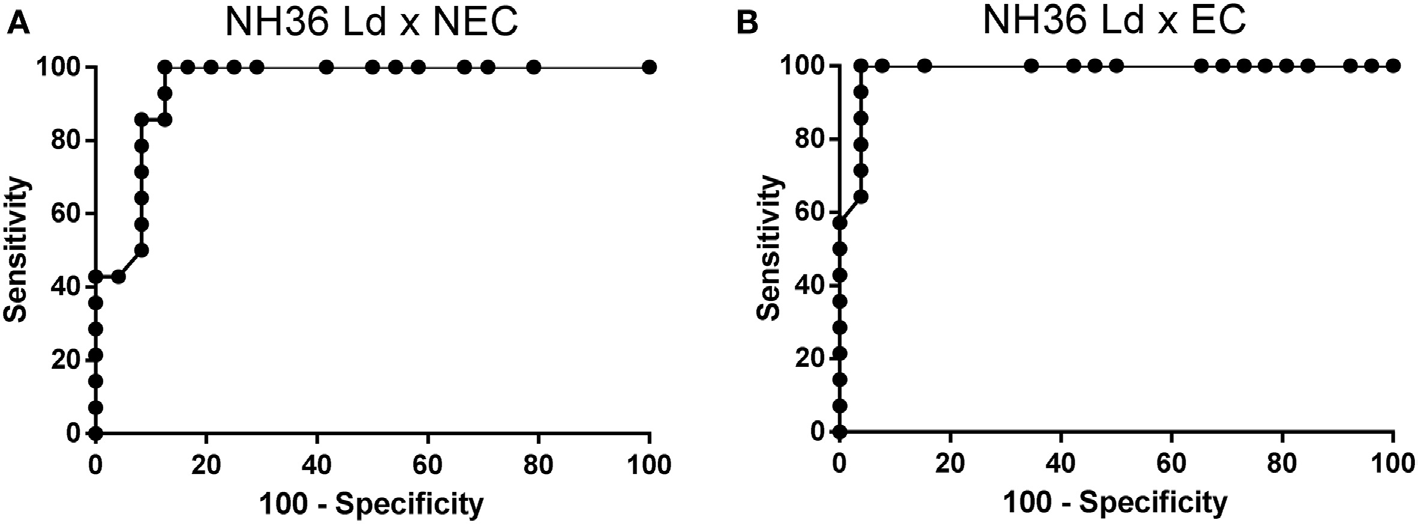
Sensitivities and specificities of NH36 ELISA for IgG3 antibodies. Receiver operating characteristics (ROC) curve for NH36-based ELISAs applied on *Leishmania (Leishmania) donovani* visceral leishmaniasis cases versus **(A)** NEC and **(B)** endemic controls (EC).

The accuracy of these results was confirmed by calculation of the cutoff values by the Receiver operating characteristic (ROC) analysis and the Youden test (Table 2). In both methods, the maximal accuracy values are equal to 1. In fact, NH36 discriminated the sera of *L. (L.) donovani* infection from controls with the highest accuracy values (0.9 < AUC–ROC ≤ 1) (Table 2). Sera of *L. (L.) infantum* infection, In contrast, were separated from sera of non-endemic area with moderate accuracy values (0.7 < AUC–ROC ≤ 0.89) and from sera of endemic area controls with low accuracy values (0.5 < AUC–ROC ≤ 0.69) (Table 2). As illustrated in Figure 5, the sensitivities and specificities obtained in the NH36 ELISA against samples of *L. (L.) donovani*-infected patients were maximal and gave the best receiver operating characteristic (ROC) curves. Calculation of cutoff values by Youden test also confirmed that NH36 displays the best performance against sera of VL patients infected with *L. (L.) donovani* (0.8030 and 0.8510) (Table 2).

**Table 2:** Cross-species IgG3 reactivity of the NH36 antigen.

Comparisons	Cut-off ROC	AUC	Cut-off Youden	Youden index
NH36 VL Ld × NEC	0.0315	0.9479	0.0280	0.8030
NH36 VL Ld × EC	0.0300	0.9849	0.0270	0.8510
NH36 VL Li × NEC	0.0180	0.7100	0.0280	0.3030
NH36 VL Li × EC	0.0205	0.6484	0.0270	0.3510

F1 VL Ld × NEC	0.0185	0.8661	0.0100	0.5770
F1 VL Ld × EC	0.0200	0.8255	0.0130	0.6320
F1 VL Li × NEC	0.0170	0.8624	0.0100	0.4500
F1 VL Li × EC	0.0200	0.8087	0.0130	0.5040

*VL Ld, visceral leishmaniasis caused by infection with Leishmania (Leishmania) donovani; VL Li, visceral leishmaniasis caused by infection with Leishmania (L.) infantum*.

In contrast, and different from what observed for NH36, F1 recognized the sera of individuals infected with either *L. (L.) donovani* or *L. (L.) infantum*, with similar moderate ROC–AUC values (0.8087–0.8661) (Table 2) and with lower and similar Youden indexes than NH36.

**Figure 6:**
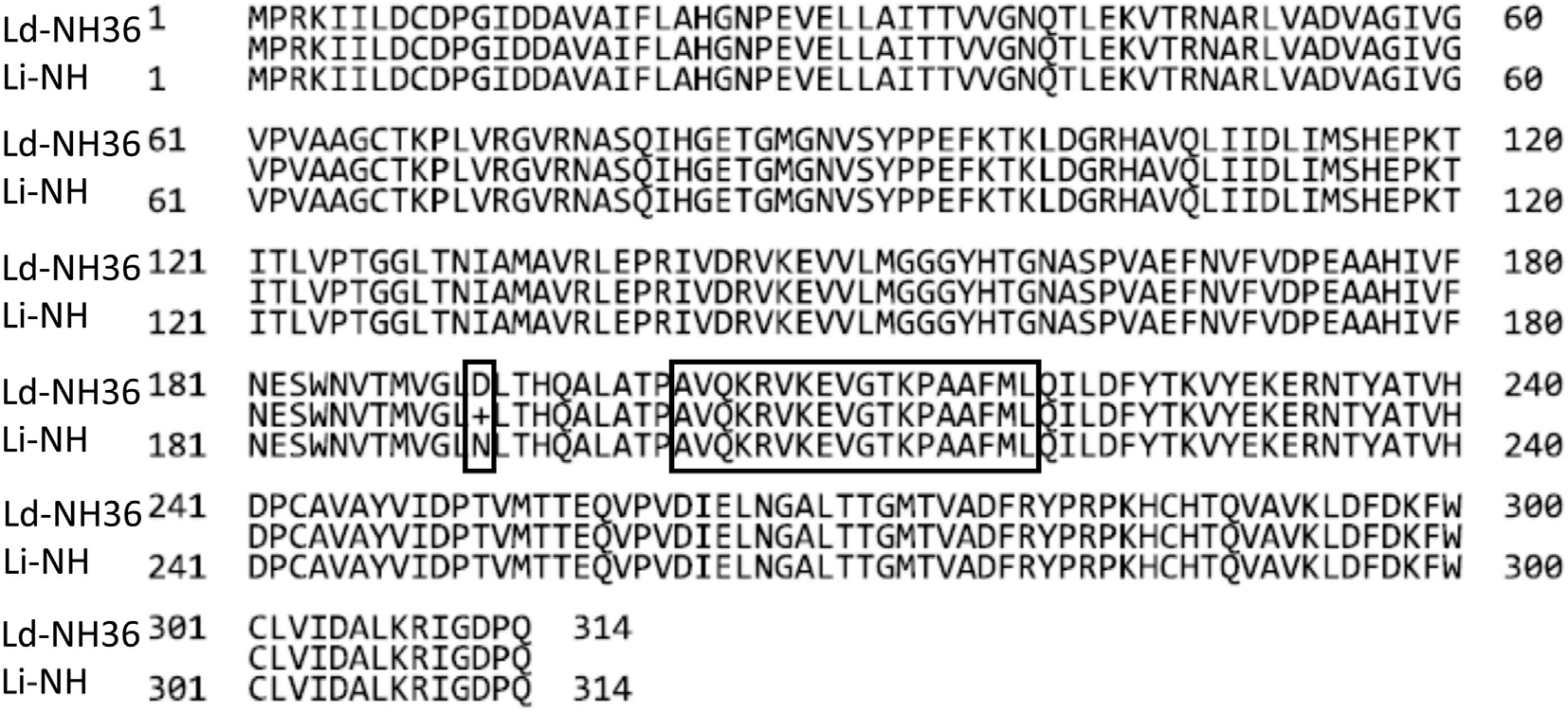
Sequence analysis of nucleoside hydrolases of *Leishmania (Leishmania) donovani* and *Leishmania (Leishmania) infantum*. The sequences of the nucleoside hydrolases NH36 of *L. (L.) donovani* AAG02281.1 (Ld-NH36) and *L. (L.) infantum* AMP43260.1 and CA F05930.1 (Li-NH) were aligned using the BLASTP GenBank program. The line in the middle of the two sequences shows the amino acids shared by the two NHs. The peptide sequence of the strongest B-cell epitope of F3 (position 202–219) (22) and the substitution of aspartic acid in Ld-NH36 for asparagine in Ld-NHs are marked by empty black squares.

We investigated further the possible reason for the higher antigenicity of NH36 against the sera of patients infected with *L. (L.) donovani*. Figure 6 summarizes the sequence homology and alignment found between the NH36 of *L. (L.) donovani*, and two reported sequences of NH of *L. (L.) infantum*. The analysis revealed 99% of homology with differences in only one amino acid: aspartic acid in NH36 of *L. (L.) donovani* was substituted by asparagine in NHs of *L. (L.) infantum*.

## Discussion

F1 is the main domain of NH36 recognized by the lymphoproliferative response of *L. (L.) infantum*-cured and asymptomatic individuals. In the asymptomatic subjects, the F1 domain induced a 19% higher stimulation index than NH36 domain. This result confirmed its antigenic predominance in acquired resistance to the disease. Coincidentally and supporting its cross-protective capabilities, we recently described that the F2 together with F1 was the strongest immunogenic domains of NH36 that were recognized by asymptomatic and cured VL patients infected with *L. (L.) infantum chagasi* in North East Brazil (28). In fact, F1 promoted a higher secretion of IFN-γ, IL-1β, IL-17, IL-12p70, and TNF-α in these patients than in endemic area controls (28).

In this investigation, we show that the lymphoproliferative responses to F1 and NH36 of cured/exposed *L. (L.) infantum*-infected subjects were accompanied by a specific IFN-γ secretion. Furthermore, the levels of IFN-γ and TNF-α and the IFN-γ/IL-10 and TNF-α/IL-10 ratios were higher in response to F1 than to NH36 in cured VL patients, suggesting the triggering of a Th1 response probably due to the F1 domain epitopes. Noteworthy, the SLA complex antigen induced levels of TNF-α not different from NH36 and F1, in asymptomatic subjects, and lower than NH36, in cured patients. In addition, IL-17A was secreted by asymptomatic and cured CL patients, in response to F1. The SLA control promoted a much lower secretion of TNF-α and IL-17A. These findings correlate with our previous observations showing that NH36 and F1 domains are strong inducers of the Th1 response. Both vaccines indeed promote increased frequencies of CD4^+^ T cells secreting IL-2, TNF-α, and both TNF-α and IL-2 together (22–24), which are, according to the CD4^+^ T-cell differentiation model proposed by Seder et al. (38), the reservoirs of memory CD4^+^ T cells that also exhibit effector potential.

Our results support the recent report of Santos et al. (28) who described a predicted epitope for CD4^+^ T cells in F1, which is highly conserved in the *Leishmania* genus and is highly promiscuous (amino acids 64–93, AAGCTKPLVRGVRNASQIHG). This epitope shows 80% probabilities of molecular binding to 18/25 HLA-DR most common molecules (28). The composition of this epitope is identical in the species that cause visceral leishmaniasis [*L. (L.) donovani, L. (L.) infantum*, and *L. (L.) infantum chagasi*] (28). Therefore, our data support the evidence raised by Mauricio et al. (39, 40) that consider *L. (L.) infantum* and *L. (L.) infantum chagasi* as the same species.

In addition, as markers of VL resistance, the F2 together with F1 induced the strongest secretion of IL-17, IL-6, and IL-10 in asymptomatic DTH+ and cured human subjects infected with *L. (L.) infantum chagasi* in Brazil (28). Consistent with this, the IFN-γ increase was correlated with decreased spleen and liver sizes and with increased hematocrit counts, in response to F1 domain (28). Furthermore, IL-17 increases were associated with decreased spleen and liver sizes in response to F1 (28).

Vaccination with NH36 has proven to protect against murine cutaneous and visceral leishmaniasis (19–24) and canine visceral leishmaniasis (26, 27), through an IFN-γ-CD4 response (21, 24). Preclinical assays with F1sap vaccine have demonstrated the induction of a cellular protective Th1 immune response characterized by the secretion of IFN-γ by CD4^+^ triple-cytokine producers and the secretion of IFN-γ and TNF-α into the splenocyte supernatants (24). Some antigens have been previously proposed as potential synthetic vaccines against leishmaniasis, like KMP-11 recognized by human CD8^+^ lymphocytes and by many different HLA receptors (41), or Leish110f fusion protein (42). Our results suggest that F1 domain of *L. (L.) donovani*-NH36 may be consequently involved in the generation of a protective response against *L. (L.) infantum* human infection and represents a potential vaccine candidate for human leishmaniasis by itself, or in combination with other HLA epitopes/antigens.

Furthermore, an increment in IL-17A secretion was observed in response to NH36 in PBMC from cured/exposed *Leishmania*-infected subjects and to F1 in cured CL and asymptomatic subjects. During the leishmaniasis epidemics in Fuenlabrada, all the infections were caused by *L. (L.) infantum*, as confirmed by PCR analysis, but the main reservoirs were hares and not dogs (1). Two-third of the cases corresponded to cutaneous leishmaniasis, and only one-third of the cases corresponded to visceral leishmaniasis. A total of 15.2% of the patients were foreigners, born in Sub-Saharan Africa. Among them, visceral leishmaniasis was predominant, while in patients from Spain, cutaneous leishmaniasis was the most frequent outcome (1, 43). In addition, near Madrid, in the past, the number of human cases has never been so high and the infection was more prevalent in children and in immunocompromised adults. In this outbreak, in contrast, only 14.1% of the affected were children and 15% were immunocompromised individuals (43). The probable genetic background basis of the different outcomes should be further investigated. An increase in IL-17 secretion has been previously reported in exposed but healthy, resistant subjects who did not develop VL (44). An increase in IL-17A after PBMC stimulation has been also described in cutaneous leishmaniasis caused by *L. (L.) braziliensis* (45). The mechanisms by which IL-17 mediates protection are not understood, but it has been recently described that IL-17A and IFN-γ synergistically promote *L. (L.) infantum* killing (46). An increase in IL-17A and IFN-γ after SLA incubation in asymptomatic and cured VL solid organ transplant patients has been previously reported (32). Here, we also described that PBMC from cured/exposed *L. (L.) infantum*-infected subjects after NH36 and F1 stimulations produced both IL-17A and IFN-γ and there are no changes in the secretion of IL-10. The IL-17A response against F1 was higher in asymptomatic subjects, disclosing its relationship with the resistance to the disease.

Furthermore, the protection elicited by IL-17 against infection is associated with the downregulation of regulatory T cells and IL-10 production, while benefiting the Th1 response and improving the leishmanicidal activity of macrophages in an NO-dependent manner (46). As widely reported for IFN-γ, a protective role of IL-17 has been described to induce in vaccine-induced immunity (44). These results reinforce the potential of F1 as a vaccine to protect against *L. (L.) infantum* human infection.

Our results also showed that supernatants stimulated with F1 or NH36 from *L. (L.) donovani* increased the levels of granzyme B in cured individuals from *L. (L.) infantum* cutaneous and visceral infection. It has been previously described the ability of some peptides from *L. major* to elicit the production of granzyme B (47). Granzyme B is secreted mainly from CD8^+^ cytotoxic lymphocytes and natural killer cells and is one of the primary molecules in effector cell-mediated killing. Our results support the description of the FLAHGNPEV- (amino acids 20–28 HLA-A and B score 28 A*02:01) and YPPEFKTKL (score 21 B*0702)-predicted epitopes for CD8^+^ T cells in the F1 sequence (28). Both epitopes show identical sequence in *L. (L.) donovani* and *L. (L.) infantum* species (28) and induced the secretion of IFN-γ by PBMC of asymptomatic, *L. (L.) infantum chagasi*-infected subjects (28). In agreement with our results, F1 increased the CD8^+^IL-2^+^, CD8^+^IL-2^+^TNF-α^+^, and CD8^+^IL-2^+^TNF-α^+^IFN-γ^+^ T-cell frequencies of *L. (L.) infantum chagasi* VL patients that correlated with increased spleen and liver sizes and decreased hemoglobin and hematocrit values (28). Therefore, while cure and acquired resistance to VL correlated to the CD4^+^ Th1 and Th17 T-cell responses to F1, clinical VL outcomes, in *L. (L.) infantum chagasi*-infected individuals in contrast, correlated to CD8^+^ T-cell responses against F1, potentially involved in control of the early infection (28).

In addition, we recently observed that the predicted epitopes for CD8^+^ T cells of F1 induced *in vitro* stimulating activity on both the CD4^+^ and the CD8^+^ T-cell populations in mice vaccinated against cutaneous leishmaniasis. The F1 epitope (92–100, YPPEFKTKL) promoted simultaneously the highest frequencies of CD4^+^IFN-γ^+^TNF-α^+^, CD8^+^TNF-α^+^, and CD8^+^IFN-γ^+^ T cells (25). This sequence could also be a PADRE epitope since the synthetic more preferably PADRE peptide composition is aKXVAAWTLKAAa (48). In fact, YPPEFKTKL contains Y as the R2 residue, four residues in R3, where 3–5 amino acids are needed, and the sequences KT followed by TKL in R4, while the expected combinations for R4 are KT, TLK, or WTLK (40). This evidence strengthens the relevance of the YPPEFKTKL epitope and partially explains the immunogenic potency of the F1 domain.

Recently, the involvement of CD4^+^ T cells producing granzyme B was described in the immune response against *Leishmania* antigens (47). F1 has also two epitopes for CD4^+^, and this could be related with the high amount of granzyme B produced after stimulation, with similar levels to those induced by SLA. Furthermore, a strong correlation has been observed between IFN-γ and granzyme B productions in response to *L. (L.) major*-excreted/secreted proteins and SLA (32, 47). While we found similar figures after SLA stimulation, F1 and NH36 produced an increase in both IFN-γ and granzyme B in cured patients and IFN-γ in asymptomatic individuals. Granzyme B has recently suggested as a new marker for cured/exposed *Leishmania*-infected subjects (49), and stimulation with F1 and NH36 could also help to potentially discriminate between cured and asymptomatic individuals exposed to *L. (L.) infantum* (49).

Regarding the humoral response, the IgG3 antigen-specific humoral response is decreased after successful VL treatment and has diagnostic value in *L. (L.) donovani*-infected patients from India (50). In our investigation, increased levels of IgG3 human antibodies directed against NH36 and F1 allowed the differentiation of clinical status. Although with relatively low absorbance values, both antigens were able to significantly discriminate sera of active disease from controls. NH36 was 55% more potent in the recognition of sera from patients infected with *L. (L.) donovani* than those infected with *L. (L.) infantum*, indicating a species specificity of this antigen for humoral immune responses. In addition, in patients infected with *L. (L.) donovani*, the absorbance values directed against NH36 were higher than those directed against F1, indicating that the most potent B epitopes are not located in F1 but probably in other NH36 domains. In agreement with that, we described, in 2010, the sequences of the NH36 epitopes for antibodies predicted by the Protean Pad program (22). Two predicted epitopes belong to the F1 domain (amino acids 40–57, NQTLEKVTRNARLVADVAG and 94–108, PEFKTKLDGRHAVQLDGRHAVQL), one was located in F2 domain (114–126, MSHEPKTITLVPT), and three epitopes correspond to the F3 domain (202–219, AVQKRVKEVGTKPAAFML, IEDB 139695; 228–239, VYEKERNTYATV, IEDB 139948; 278–291, FRYPRPKHCHTQVA, IEDB 139752) (22). In the same work, we demonstrated that the three epitopes of F3 developed the strongest reactivity against the sera of healthy dogs vaccinated with Leishmune^®^ (22).

Confirming our previous results, in 2012, two linear B-cell-predicted epitopes for dog and human antibodies were identified along the sequence of NH36 (51). One of them, called peptide 18 (TTVVGNQTLEKVT), is located in the F1 domain, overlaps with the single antibody epitope identified by our group before (NQTLEKVTRNARLVADVAG) (22), and displayed high sensitivities and specificities in the diagnosis of canine and human cases of VL caused by *L. (L.) infantum chagasi* in Brazil (51). F1 was also the domain mostly recognized by sera of *L. (L.) infantum chagasi*-infected dogs, after immunotherapy with the NH36 DNA vaccine (26). The second epitope called peptide 17 (TPAVQKRVKEVGTKP) (51) belongs to the F3 domain and overlaps with the epitope AVQKRVKEVGTKPAAFML described by our group before, which induced the strongest inhibition of binding of anti-NH36 antibodies (31.4%) of dogs vaccinated with Leishmune^®^ (22). Peptide 17 showed 100% sensitivity and 94% specificity in the diagnosis of human cases of VL due to *L. (L.) infantum chagasi* infection (51).

The finding of the most antigenic B-cell epitopes in the F3 domains of NH36 (22) explain why, in this investigation, NH36 was a more potent antigen than the F1 domain alone, mainly in the detection of antibodies raised by *L.(L.) donovani* infection.

In addition, in our investigation, a species specificity was noted in the reactivity of NH36 with IgG3 of human sera. In fact, we demonstrated that the sera of patients infected with *L. (L.) donovani* reacted more with the NH36 antigen of *L. (L.) donovani* than the sera of patients infected with *L. (L.) infantum*. This species specificity was, however, not detected against the F1 domain, which was equally recognized by the sera of patients from Ethiopia and Spain; therefore, its basis would be related to the epitopes of other NH36 domains. To investigate this possibility, the sequences of NHs of the two parasites were aligned. They share 99% of identity, the only difference being one amino acid located at position 192. Indeed, aspartic acid in NH36 of *L. (L.) donovani* is substituted by asparagine in NH of *L. (L.) infantum*. Aspartic acid is a charged amino acid residue, while asparagine is a non-charged but polar amino acid. Conformational B epitopes are enriched in charged and polar amino acids (51, 52). The importance of the substitution of aspartic acid for asparagine in position 192 might be enhanced by the fact that only nine residues separate it from the most immunogenic epitope of F3, which is responsible for 31% of the inhibition of binding of NH36 to antibodies of Leishmune^®^-vaccinated dogs (202–219, AVQKRVKEVGTKPAAFML) (22). Therefore, the substitution of one charged amino acid in the vicinity of the most important epitope of F3 might explain the higher antigenicity found in NH36 of *L. (L.) donovani*. Our assumption of the importance of this area is supported by the description of the predicted epitope 17, also in this region, but which starts at amino acid 200 (51), instead of 202 (22).

In contrast, the similar AUC and Youden index values determined by F1 in the assay of sera of patients infected with *L. (L.) donovani* and *L. (L.) infantum* might indicate the importance of the predicted epitopes for antibodies in the sequence of F1 (amino acids 40–57, NQTLEKVTRNARLVADVAG and 94–108, PEFKTKLDGRHAVQLDGRHAVQL) (22), which are completely conserved in the NHs of the two *Leishmania* species.

It is worth to note that the presence of anti-NH36 antibodies in sera of human and dog patients infected with *L.(L.) infantum chagasi* was previously demonstrated (51, 53, 54). In this article, we described for the first time the recognition of NH36 by antibodies generated by human infections with *L. (L.) infantum* and *L. (L.) donovani*.

Our data demonstrates that F1 peptide induces a recall cellular response similar or higher to the one induced by NH36, a recombinant antigen that has protected mice from infection by several *Leishmania* species and dogs from *L. (L.) infantum chagasi* infection. No human vaccine against *Leishmania* is available thus far. The F1 domain of *L. (L.) donovani*-NH36, which contributes to protection against human infection by *L. (L.) infantum chagasi* (28), may also be involved in the generation of a protective response against *L. (L.) infantum* infection and represents a potential universal vaccine candidate for the control of human visceral leishmaniasis alone, or in combination with other HLA epitopes/antigens.

## Ethics Statement

This study was approved by the Hospital de Fuenlabrada (Madrid) Ethics and Research Committee (APR 12–65 and APR 14–64). In addition, the protocols for the collection of sera samples of healthy blood donors of the Hospital Universitário Clementino Fraga Filho (HUCFFo)-Blood Transfusion service of the Universidade Federal do Rio de Janeiro (Rio de Janeiro, Brazil) were approved by the Comité de Ética em Pesquisa of the HUCFFo-Faculdade de Medicina-UFRJ, Brazil, according to the GM-MS 158-2016 regulation of the Brazilian Ministry of Health. All participants gave their written informed consent.

## Author Contributions

LF, AI-M, and DN conducted the experiments; LF, AI-M, MS, DN, and CC acquired the data; LF, AI-M, and CP-d-S analyzed the data; CP-d-S, RA, PL, and JM designed the research studies; EC and JM provided reagents; EC and CP-d-S wrote the manuscript. All authors have read and approved the final manuscript.

## Conflict of Interest Statement

DN and CP-d-S are the inventors of the patent file PI1015788-3 (INPI Brazil). EC, LF, AI-M, MS, PL, CC, RA, and JM declare no conflict of interest.
